# Supplementary material for: A Likelihood Approach for Real-Time Calibration of Stochastic Compartmental Epidemic Models
Source: PLoS Comput Biol. 2017 Jan 17;13(1):e1005257. doi: 10.1371/journal.pcbi.1005257 (PMC5240920; doi:10.1371/journal.pcbi.1005257)
Supplement: S1 File — (TAR.GZ) [file pcbi.1005257.s014.tar.gz › HSPH_Online-SI-Revision/MSS10/n100-severe/n100-severe_NEW2_table8.pdf]

1	newly 3 weeks spec	405.	925.62	{875., 975.}	128.548	0.358585	newly 3 weeks cumul	1885.	4037.54	{3960., 4111.}	114.193	0.330746	newly 5 weeks spec	212.	457.46	{418., 501.}	115.783	0.332908	newly 5 weeks cumul	2436.	5148.8	{5021., 5290.}	111.363	0.324945	newly 42 weeks cumul	2940.	6208.99	{6032., 6402.}	111.19	0.324545
2	newly 3 weeks spec	32.	13.42	{2., 29.}	62.625	0.623894	newly 3 weeks cumul	144.	92.09	{34., 141.}	40.2569	0.270834	newly 5 weeks spec	7.	2.93	{0., 8.}	75.8571	1.03561	newly 5 weeks cumul	163.	101.48	{34., 160.}	42.589	0.292451	newly 49 weeks cumul	163.	103.88	{34., 165.}	42.7853	0.291055
3	newly 3 weeks spec	20.	6.54	{1., 12.}	67.8	0.704455	newly 3 weeks cumul	87.	49.48	{25., 75.}	43.8851	0.287032	newly 5 weeks spec	9.	1.77	{0., 6.}	81.6667	1.26581	newly 5 weeks cumul	101.	54.29	{25., 84.}	46.4851	0.315437	newly 50 weeks cumul	112.	55.06	{25., 88.}	50.8393	0.35449
4	newly 3 weeks spec	173.	433.46	{398., 469.}	150.555	0.397962	newly 3 weeks cumul	880.	2177.27	{2102., 2273.}	147.417	0.393247	newly 5 weeks spec	105.	176.52	{148., 208.}	68.1143	0.22225	newly 5 weeks cumul	1112.	2631.91	{2519., 2748.}	136.683	0.373938	newly 48 weeks cumul	1341.	2941.07	{2796., 3072.}	119.319	0.340731
5	newly 3 weeks spec	780.	877.94	{820., 929.}	12.5564	0.0509174	newly 3 weeks cumul	2856.	4071.73	{3981., 4160.}	42.5676	0.153956	newly 5 weeks spec	402.	398.34	{355., 439.}	6.1791	0.0271399	newly 5 weeks cumul	3744.	5060.36	{4913., 5171.}	35.1592	0.130761	newly 42 weeks cumul	4829.	5865.59	{5673., 6037.}	21.4659	0.0843238
6	newly 3 weeks spec	252.	355.	{316., 394.}	40.873	0.147291	newly 3 weeks cumul	1205.	1759.6	{1678., 1847.}	46.0249	0.164128	newly 5 weeks spec	97.	150.48	{126., 177.}	55.134	0.1868	newly 5 weeks cumul	1441.	2141.02	{2044., 2257.}	48.5788	0.171578	newly 48 weeks cumul	1575.	2408.24	{2272., 2582.}	52.9041	0.183931
7	newly 3 weeks spec	63.	73.62	{57., 96.}	23.1111	0.087832	newly 3 weeks cumul	435.	454.32	{397., 512.}	9.42989	0.0393753	newly 5 weeks spec	20.	22.39	{12., 32.}	32.65	0.131935	newly 5 weeks cumul	497.	517.35	{448., 589.}	9.87726	0.0413868	newly 49 weeks cumul	509.	542.76	{464., 631.}	11.5914	0.0475238
8	newly 3 weeks spec	292.	153.39	{121., 182.}	47.4692	0.285823	newly 3 weeks cumul	1253.	793.76	{689., 875.}	36.6512	0.200189	newly 5 weeks spec	140.	61.81	{42., 82.}	55.85	0.367928	newly 5 weeks cumul	1606.	952.05	{828., 1068.}	40.7192	0.229178	newly 45 weeks cumul	1911.	1051.65	{892., 1198.}	44.9686	0.262212
9	newly 3 weeks spec	0.1	2.99	{0., 8.}	2982.	0.865021	newly 3 weeks cumul	4.	28.94	{7., 54.}	627.	0.769428	newly 5 weeks spec	0.1	0.49	{0., 2.}	564.	0.188713	newly 5 weeks cumul	4.	30.39	{7., 61.}	663.25	0.782603	newly 50 weeks cumul	4.	30.78	{7., 61.}	673.	0.786399
10	newly 3 weeks spec	115.	62.15	{43., 81.}	45.9739	0.280887	newly 3 weeks cumul	507.	362.19	{293., 427.}	28.5621	0.150433	newly 5 weeks spec	26.	20.11	{9., 32.}	32.9615	0.189133	newly 5 weeks cumul	597.	416.5	{330., 493.}	30.2345	0.161034	newly 43 weeks cumul	631.	444.16	{358., 532.}	29.6101	0.157498
11	newly 3 weeks spec	53.	34.42	{19., 50.}	37.2075	0.228018	newly 3 weeks cumul	267.	226.1	{159., 285.}	19.1685	0.0975371	newly 5 weeks spec	7.	9.12	{1., 17.}	74.	0.344282	newly 5 weeks cumul	288.	254.04	{180., 322.}	18.0972	0.0900794	newly 49 weeks cumul	300.	264.14	{186., 331.}	18.4933	0.0926184
12	newly 3 weeks spec	467.	1465.13	{1405., 1531.}	213.732	0.496351	newly 3 weeks cumul	2017.	6019.27	{5922., 6103.}	198.427	0.474806	newly 5 weeks spec	256.	802.7	{762., 838.}	213.555	0.49603	newly 5 weeks cumul	2654.	7902.65	{7762., 8037.}	197.764	0.473835	newly 42 weeks cumul	3292.	10 115.2	{9926., 10 330.}	207.267	0.487463
13	newly 3 weeks spec	476.	520.99	{477., 567.}	10.1996	0.0416462	newly 3 weeks cumul	2172.	2270.51	{2182., 2349.}	4.72422	0.0198979	newly 5 weeks spec	224.	263.93	{235., 292.}	18.2277	0.0714101	newly 5 weeks cumul	2758.	2902.79	{2777., 3019.}	5.39123	0.0226333	newly 42 weeks cumul	3330.	3524.47	{3359., 3682.}	6.16486	0.0257563
14	newly 3 weeks spec	744.	973.24	{908., 1036.}	30.8118	0.11611	newly 3 weeks cumul	3264.	4192.54	{4060., 4319.}	28.4479	0.108605	newly 5 weeks spec	407.	488.71	{444., 536.}	20.1892	0.0787785	newly 5 weeks cumul	4260.	5377.3	{5182., 5563.}	26.2277	0.100993	newly 42 weeks cumul	5829.	6560.71	{6310., 6832.}	12.5529	0.0511517
15	newly 3 weeks spec	170.	147.67	{107., 189.}	17.0529	0.0845864	newly 3 weeks cumul	891.	757.04	{636., 871.}	15.8182	0.0775506	newly 5 weeks spec	60.	55.55	{34., 76.}	23.8833	0.116323	newly 5 weeks cumul	1068.	905.08	{753., 1050.}	16.1311	0.0792535	newly 44 weeks cumul	1164.	997.56	{797., 1160.}	15.4966	0.0763225
16	newly 3 weeks spec	648.	334.34	{295., 372.}	48.4043	0.289104	newly 3 weeks cumul	2744.	1392.01	{1303., 1480.}	49.2708	0.2953	newly 5 weeks spec	394.	183.42	{155., 218.}	53.4467	0.335884	newly 5 weeks cumul	3600.	1823.76	{1687., 1949.}	49.34	0.295995	newly 43 weeks cumul	4481.	2328.41	{2126., 2497.}	48.0382	0.285196
17	newly 3 weeks spec	33.	53.61	{30., 78.}	69.3636	0.216305	newly 3 weeks cumul	309.	325.58	{231., 423.}	19.1909	0.0813016	newly 5 weeks spec	37.	15.44	{5., 28.}	58.4865	0.478732	newly 5 weeks cumul	396.	370.25	{258., 486.}	18.1692	0.0859718	newly 49 weeks cumul	405.	391.77	{271., 514.}	18.3975	0.0842695
18	newly 3 weeks spec	122.	171.21	{136., 201.}	40.6475	0.144121	newly 3 weeks cumul	687.	865.56	{767., 949.}	26.0204	0.0992067	newly 5 weeks spec	37.	70.31	{46., 91.}	90.1892	0.264285	newly 5 weeks cumul	830.	1045.52	{910., 1172.}	25.9663	0.0985611	newly 47 weeks cumul	928.	1165.04	{995., 1321.}	25.5668	0.0963823
19	newly 3 weeks spec	77.	46.83	{31., 65.}	39.3636	0.237241	newly 3 weeks cumul	381.	269.71	{225., 327.}	29.3097	0.155412	newly 5 weeks spec	32.	16.07	{7., 28.}	50.5313	0.374077	newly 5 weeks cumul	445.	311.71	{258., 372.}	30.1191	0.160843	newly 47 weeks cumul	467.	334.17	{267., 410.}	29.0086	0.154605
20	newly 3 weeks spec	99.	64.98	{50., 80.}	34.5051	0.191888	newly 3 weeks cumul	531.	368.3	{322., 424.}	30.6403	0.161827	newly 5 weeks spec	19.	22.06	{10., 34.}	41.1579	0.166395	newly 5 weeks cumul	601.	429.42	{367., 489.}	28.5491	0.149561	newly 45 weeks cumul	622.	462.32	{385., 540.}	25.7621	0.133487
21	newly 3 weeks spec	24.	38.55	{20., 54.}	67.9583	0.213216	newly 3 weeks cumul	229.	246.82	{183., 308.}	16.1223	0.0656341	newly 5 weeks spec	14.	10.18	{2., 19.}	48.	0.354721	newly 5 weeks cumul	254.	277.51	{209., 353.}	17.626	0.0706398	newly 49 weeks cumul	256.	288.59	{215., 374.}	20.0352	0.0780198
22	newly 3 weeks spec	57.	54.39	{27., 73.}	23.7368	0.120056	newly 3 weeks cumul	329.	324.11	{226., 411.}	16.5198	0.0744395	newly 5 weeks spec	27.	16.46	{7., 29.}	45.7037	0.325534	newly 5 weeks cumul	392.	369.92	{262., 472.}	16.8265	0.0791724	newly 47 weeks cumul	426.	386.93	{273., 481.}	17.6362	0.0858178
23	newly 3 weeks spec	0.1	1.84	{0., 5.}	1840.	0.728111	newly 3 weeks cumul	12.	16.06	{4., 28.}	68.8333	0.272738	newly 5 weeks spec	0.1	0.22	{0., 0.}	306.	0.0987506	newly 5 weeks cumul	12.	16.75	{4., 32.}	74.25	0.282033	newly 51 weeks cumul	12.	16.81	{4., 32.}	74.75	0.282723
24	newly 3 weeks spec	10.	11.29	{3., 20.}	57.9	0.315057	newly 3 weeks cumul	103.	88.27	{53., 128.}	24.9029	0.131555	newly 5 weeks spec	0.1	2.17	{0., 7.}	2158.	0.823887	newly 5 weeks cumul	103.	96.1	{53., 139.}	24.9709	0.12436	newly 52 weeks cumul	103.	98.3	{53., 139.}	25.6893	0.125594
25	newly 3 weeks spec	0.1	5.85	{0., 13.}	5790.	1.36258	newly 3 weeks cumul	33.	58.1	{20., 97.}	92.6667	0.296635	newly 5 weeks spec	0.1	1.03	{0., 4.}	1084.	0.351865	newly 5 weeks cumul	33.	61.36	{22., 106.}	102.303	0.312469	newly 52 weeks cumul	33.	61.96	{22., 106.}	104.121	0.31479
26	newly 3 weeks spec	105.	84.6	{65., 103.}	21.0286	0.107365	newly 3 weeks cumul	559.	495.4	{444., 544.}	11.7424	0.0555577	newly 5 weeks spec	34.	27.36	{16., 38.}	28.9412	0.160667	newly 5 weeks cumul	657.	572.08	{499., 645.}	13.3577	0.0639771	newly 46 weeks cumul	704.	608.99	{517., 691.}	14.4446	0.0696712
27	newly 3 weeks spec	67.	48.56	{31., 67.}	30.3582	0.17246	newly 3 weeks cumul	373.	286.18	{224., 346.}	24.0536	0.124813	newly 5 weeks spec	19.	13.77	{4., 24.}	41.6316	0.308844	newly 5 weeks cumul	421.	326.07	{251., 399.}	23.6793	0.123152	newly 47 weeks cumul	444.	342.67	{254., 435.}	24.1689	0.126829
28	newly 3 weeks spec	219.	170.08	{136., 202.}	22.3744	0.114669	newly 3 weeks cumul	956.	815.87	{720., 907.}	14.6579	0.0704323	newly 5 weeks spec	119.	78.15	{54., 102.}	34.6471	0.195203	newly 5 weeks cumul	1227.	1010.27	{875., 1143.}	17.6634	0.0863605	newly 46 weeks cumul	1619.	1157.59	{997., 1327.}	28.4997	0.148201
29	newly 3 weeks spec	122.	41.52	{26., 56.}	65.9672	0.486381	newly 3 weeks cumul	698.	233.67	{184., 278.}	66.5229	0.480603	newly 5 weeks spec	37.	14.09	{5., 25.}	61.9189	0.50523	newly 5 weeks cumul	790.	272.14	{208., 329.}	65.5519	0.469005	newly 47 weeks cumul	845.	289.23	{214., 358.}	65.7716	0.472885
30	newly 3 weeks spec	189.	197.31	{155., 236.}	13.3386	0.0566051	newly 3 weeks cumul	1013.	956.55	{849., 1064.}	7.98717	0.0366349	newly 5 weeks spec	86.	83.91	{57., 114.}	18.8488	0.0851493	newly 5 weeks cumul	1247.	1167.33	{1015., 1313.}	8.80433	0.0406927	newly 43 weeks cumul	1455.	1321.09	{1141., 1485.}	10.5835	0.050073
31	newly 3 weeks spec	1.	2.22	{0., 8.}	240.	0.80732	newly 3 weeks cumul	19.	19.06	{5., 40.}	55.3684	0.278454	newly 5 weeks spec	0.1	0.33	{0., 0.}	418.	0.0930449	newly 5 weeks cumul	19.	19.97	{5., 41.}	58.5789	0.282942	newly 52 weeks cumul	19.	20.24	{5., 41.}	60.	0.286129
32	newly 3 weeks spec	28.	16.42	{5., 29.}	46.2143	0.333493	newly 3 weeks cumul	154.	125.77	{84., 172.}	23.9545	0.126237	newly 5 weeks spec	6.	3.87	{0., 10.}	70.5	0.735924	newly 5 weeks cumul	176.	137.83	{88., 185.}	26.3693	0.142335	newly 50 weeks cumul	190.	141.06	{88., 189.}	29.0421	0.161355
33	newly 3 weeks spec	32.	14.42	{5., 23.}	54.9375	0.41328	newly 3 weeks cumul	204.	89.22	{61., 124.}	56.2647	0.373401	newly 5 weeks spec	18.	4.04	{0., 9.}	78.4444	0.950803	newly 5 weeks cumul	235.	101.13	{70., 140.}	56.966	0.382227	newly 50 weeks cumul	268.	105.52	{70., 145.}	60.6269	0.423169
34	newly 3 weeks spec	218.	96.43	{73., 123.}	55.7661	0.362422	newly 3 weeks cumul	1079.	533.93	{452., 594.}	50.5162	0.308239	newly 5 weeks spec	118.	33.04	{20., 44.}	72.	0.57428	newly 5 weeks cumul	1346.	623.49	{527., 699.}	53.6783	0.337353	newly 42 weeks cumul	1517.	674.24	{556., 762.}	55.5544	0.356021
35	newly 3 weeks spec	0.1	5.71	{0., 14.}	5642.	1.40528	newly 3 weeks cumul	24.	55.65	{24., 89.}	138.708	0.352728	newly 5 weeks spec	0.1	0.58	{0., 2.}	642.	0.259857	newly 5 weeks cumul	24.	57.91	{24., 95.}	148.125	0.367068	newly 51 weeks cumul	24.	58.65	{24., 96.}	151.208	0.370597
36	newly 3 weeks spec	53.	28.84	{15., 47.}	46.0755	0.308723	newly 3 weeks cumul	336.	204.64	{152., 259.}	39.0952	0.223661	newly 5 weeks spec	17.	7.14	{1., 14.}	62.5882	0.613547	newly 5 weeks cumul	377.	225.87	{166., 288.}	40.0875	0.231904	newly 49 weeks cumul	391.	232.17	{171., 303.}	40.6215	0.236407
37	newly 3 weeks spec	3.	7.77	{0., 19.}	211.	0.641931	newly 3 weeks cumul	35.	65.98	{20., 109.}	106.114	0.317964	newly 5 weeks spec	0.1	1.56	{0., 5.}	1590.	0.536294	newly 5 weeks cumul	35.	71.53	{20., 119.}	121.514	0.34215	newly 51 weeks cumul	35.	72.64	{20., 122.}	124.686	0.346503
38	newly 3 weeks spec	40.	66.31	{48., 83.}	66.275	0.21244	newly 3 weeks cumul	256.	414.66	{360., 469.}	61.9766	0.207154	newly 5 weeks spec	12.	20.23	{10., 30.}	76.9167	0.237163	newly 5 weeks cumul	282.	472.49	{409., 543.}	67.5496	0.221178	newly 49 weeks cumul	298.	496.81	{422., 576.}	66.7148	0.218534
39	newly 3 weeks spec	150.	122.14	{93., 152.}	20.7467	0.106325	newly 3 weeks cumul	734.	611.41	{524., 706.}	16.8351	0.082892	newly 5 weeks spec	40.	48.28	{29., 70.}	34.	0.126851	newly 5 weeks cumul	880.	736.35	{627., 871.}	16.8307	0.0830332	newly 46 weeks cumul	975.	816.93	{685., 976.}	17.3815	0.0857998
40	newly 3 weeks spec	92.	78.88	{56., 101.}	20.8043	0.107492	newly 3 weeks cumul	452.	433.77	{346., 516.}	12.0642	0.0561121	newly 5 weeks spec	39.	25.05	{10., 41.}	41.0513	0.264995	newly 5 weeks cumul	559.	502.89	{381., 609.}	14.3023	0.0697962	newly 49 weeks cumul	609.	537.2	{396., 663.}	15.9442	0.0787768
41	newly 3 weeks spec	14.	16.47	{6., 28.}	48.6429	0.207007	newly 3 weeks cumul	127.	114.23	{73., 160.}	20.9055	0.103672	newly 5 weeks spec	4.	4.57	{0., 11.}	93.75	0.590531	newly 5 weeks cumul	135.	127.91	{76., 172.}	22.1259	0.105794	newly 51 weeks cumul	136.	130.67	{76., 184.}	22.4779	0.106455
42	newly 3 weeks spec	202.	304.29	{269., 340.}	50.6386	0.175859	newly 3 weeks cumul	987.	1419.15	{1319., 1519.}	43.7842	0.157173	newly 5 weeks spec	95.	136.9	{114., 159.}	44.2105	0.155281	newly 5 weeks cumul	1233.	1760.61	{1633., 1889.}	42.7908	0.154072	newly 44 weeks cumul	1344.	2042.07	{1873., 2199.}	51.9397	0.180862
43	newly 3 weeks spec	252.	220.88	{169., 270.}	16.2222	0.0792352	newly 3 weeks cumul	1153.	1023.99	{884., 1166.}	12.209	0.0582531	newly 5 weeks spec	99.	97.09	{66., 126.}	19.3838	0.0886483	newly 5 weeks cumul	1387.	1266.07	{1085., 1444.}	10.8284	0.0510245	newly 43 weeks cumul	1536.	1462.81	{1219., 1680.}	10.1361	0.0465116
44	newly 3 weeks spec	39.	19.24	{8., 31.}	51.7949	0.362278	newly 3 weeks cumul	267.	146.01	{109., 181.}	45.3146	0.273435	newly 5 weeks spec	9.	4.4	{0., 10.}	64.6667	0.750082	newly 5 weeks cumul	301.	158.78	{114., 204.}	47.2492	0.290683	newly 50 weeks cumul	304.	162.07	{114., 207.}	46.6875	0.287289
45	newly 3 weeks spec	130.	145.62	{114., 176.}	17.3231	0.0685483	newly 3 weeks cumul	816.	805.39	{706., 901.}	7.13113	0.0314591	newly 5 weeks spec	64.	48.58	{31., 72.}	29.5625	0.163991	newly 5 weeks cumul	987.	939.6	{811., 1056.}	8.67275	0.0394734	newly 48 weeks cumul	1048.	1008.05	{867., 1140.}	8.97615	0.0405716
46	newly 3 weeks spec	72.	75.1	{55., 96.}	18.75	0.0789608	newly 3 weeks cumul	478.	442.13	{371., 515.}	11.9477	0.0555225	newly 5 weeks spec	30.	23.36	{12., 36.}	34.0667	0.194956	newly 5 weeks cumul	550.	509.36	{420., 607.}	12.28	0.0570875	newly 47 weeks cumul	566.	538.98	{447., 640.}	11.7597	0.0534957
47	newly 3 weeks spec	463.	476.67	{434., 515.}	5.81641	0.0245757	newly 3 weeks cumul	1902.	2006.71	{1920., 2086.}	5.64511	0.0236623	newly 5 weeks spec	209.	256.03	{226., 283.}	22.6268	0.0867673	newly 5 weeks cumul	2424.	2613.59	{2496., 2739.}	7.96411	0.0330272	newly 43 weeks cumul	2941.	3313.57	{3114., 3541.}	12.781	0.0517527
48	newly 3 weeks spec	166.	502.28	{465., 540.}	202.578	0.48012	newly 3 weeks cumul	968.	2518.53	{2422., 2606.}	160.179	0.415107	newly 5 weeks spec	51.	204.04	{174., 233.}	300.078	0.599577	newly 5 weeks cumul	1120.	3041.1	{2926., 3157.}	171.527	0.433628	newly 48 weeks cumul	1215.	3390.67	{3219., 3586.}	179.067	0.44539
49	newly 3 weeks spec	3.	4.12	{0., 11.}	123.333	0.742698	newly 3 weeks cumul	31.	35.59	{11., 63.}	54.0323	0.242043	newly 5 weeks spec	0.1	1.03	{0., 3.}	1080.	0.356504	newly 5 weeks cumul	32.	38.76	{11., 73.}	60.125	0.255303	newly 52 weeks cumul	32.	39.42	{11., 77.}	62.0625	0.258502
50	newly 3 weeks spec	18.	6.21	{0., 14.}	68.7222	0.881805	newly 3 weeks cumul	46.	52.62	{16., 88.}	46.3043	0.218916	newly 5 weeks spec	1.	1.34	{0., 5.}	156.	0.77308	newly 5 weeks cumul	52.	57.6	{17., 97.}	45.5769	0.223698	newly 51 weeks cumul	52.	59.06	{17., 99.}	47.7692	0.228244
